# Control of HIV Latency by Epigenetic and Non-Epigenetic Mechanisms

**DOI:** 10.2174/157016211798998736

**Published:** 2011-12

**Authors:** Uri Mbonye, Jonathan Karn

**Affiliations:** Department of Molecular Biology and Microbiology, Case Western Reserve University, Cleveland, OH 44106, USA

**Keywords:** HIV latency, Tat, P-TEFb, epigenetics, NF-κB.

## Abstract

Intensive antiretroviral therapy successfully suppresses viral replication but is unable to eradicate the virus. HIV persists in a small number of resting memory T cells where HIV has been transcriptionally silenced. This review will focus on recent insights into the HIV transcriptional control mechanisms that provide the biochemical basis for understanding latency. There are no specific repressors of HIV transcription encoded by the virus, instead latency arises when the regulatory feedback mechanism driven by HIV Tat expression is disrupted. Small changes in transcriptional initiation, induced by epigenetic silencing, lead to profound restrictions in Tat levels and force the entry of proviruses into latency. In resting memory T cells, which carry the bulk of the latent viral pool, additional restrictions, especially the limiting cellular levels of the essential Tat cofactor P-TEFb and the transcription initiation factors NF-κB and NFAT ensure that the provirus remains silenced unless the host cell is activated. The detailed understanding of HIV transcription is providing a framework for devising new therapeutic strategies designed to purge the latent viral pool. Importantly, the recognition that there are multiple restrictions imposed on latent proviruses suggest that proviral reactivation will not be achieved when only a single reactivation step is targeted and that any optimal activation strategy will require both removal of epigenetic blocks and the activation of P-TEFb.

Even with an intensive regimen of highly active antiretroviral therapy (HAART), HIV infections persist throughout the lifetimes of patients due to the existence of a latent reservoir of virus in peripheral blood and lymphoid tissues. Latent HIV proviruses quickly resume active replication when HAART is interrupted [1-3]. Although it is difficult to exclude the possibility that slowly replicating virus persists in sanctuary sites which are poorly accessed by the antiviral drugs, genetic and biochemical evidence strongly suggests that the major latent viral reservoir resides in a very small population of resting memory CD4 T cells (~1 in 10^6^ cells)[1,2, 4-16]. The residual virus recovered from treated patients [17], and the rebounding virus recovered during the short treatment interruptions [18], have much less sequence heterogeneity than would be expected for a viral population replicating at low levels.

Eliminating the latent reservoir is particularly challenging since the reservoir is established early during infection [9], is extremely stable, with an estimated half-life of 44 months [8], and can be replenished during episodes of viremia [19] or by homeostatic replacement of latently infected cells [13]. Since intensification of antiviral regimens has essentially no impact on eradicating the latent pool from the infected host [4], there is a pressing need to devise novel strategies that specifically target the latent reservoirs of replication-competent HIV [20, 21]. Once reactivated, the viral reservoirs can in principle be purged by antiviral immune responses, viral cytopathic effects, or even intensified antiretroviral therapy.

The molecular studies of HIV latency that we will review in this article have demonstrated that a complex combination of cellular events that suppress both the initiation of HIV transcription and its productive elongation are required for the establishment of latent proviruses. The multiple restrictions imposed on latent proviruses that need to be overcome suggest that efficient proviral reactivation will be achieved only if there is the simultaneous removal of multiple blocks to transcription initiation and elongation. Therefore, the development of molecular strategies to eradicate latent reservoirs of HIV demands an improved understanding of the molecular mechanisms controlling its transcriptional silencing and viral reactivation.

## ORGANIZATION OF THE HIV PROMOTER

**Fig. (1):**
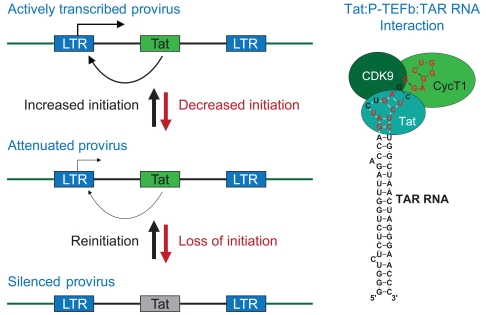
**Autoregulation of HIV transcription by Tat.** Left panels: Efficient transcription elongation from the HIV LTR is dependent upon Tat. Small changes in initiation efficiency, due to transcriptional interference or epigenetic silencing, reduce Tat levels in the cell and disproportionately inhibit transcription, driving the HIV provirus into latency. Re-initiation stimulates Tat production and restores full transcription efficiency. Right panels: Recognition of TAR RNA by Tat and P-TEFb. The red bases in TAR are recognized by Tat in the TAR bulge region, and by CycT1 in the TAR loop region.

**Fig. (2):**
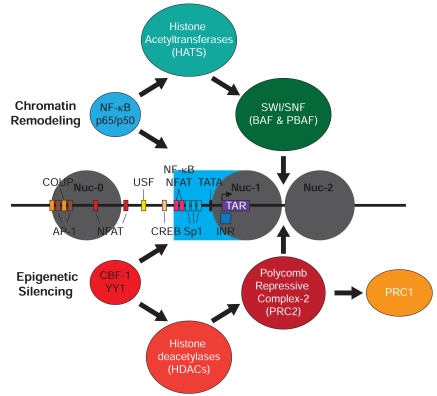
**Chromatin control of HIV transcription.** The structure of the HIV promoter and flanking nucleosomes is shown in the center. Proviral reactivation depends upon recruitment of histone acetyltransferases that are recruited to the HIV LTR following induction of NF-κB (or NFAT). The histone acetyltransferases in turn recruit the SWI/SNF chromatin remodeling machinery. This acts to remodel the restrictive nucleosome 1 (Nuc-1). During epigenetic silencing histone deacetylases are recruited to the promoter *via* DNA-binding molecules, including CBF-1 and YY1. The deacetylated proviral chromatin becomes a target for additional silencing *via* recruitment of the polycomb repressive complex-2 (PRC2) which mediates histone methylation and DNA methylation. In certain circumstances PRC2 can recruit PRC1 leading to further repression of the provirus.

HIV-1, in common with all other retroviruses uses its long terminal repeat (LTR) as the viral promoter (Fig. **1**). The HIV-1 promoter is a powerful and highly optimized transcription machine comprised of three tandem SP1 binding sites [22], an efficient TATA element [23] and a highly active initiator sequence [24]. Each of these elements participates in the co-operative binding of the initiation factor TFIID and its associated TAF co-factors to the TATA element [25] (Fig. **2**). As a result, the HIV-1 LTR is one of the most efficient promoters that has ever been studied and it is capable of supporting even higher levels of transcription than the adenovirus major late promoter or the CMV immediate early promoter.

Transcription initiation from the HIV LTR is highly inducible. In addition to the core promoter, HIV-1 utilizes a signal-responsive "enhancer region" which contains two NF-κB binding motifs [26]. Members of both the NF-κB family [27] and NFAT [28] can bind to the HIV-1 NF-κB motifs. Because their recognition sequences overlap, binding of these factors is mutually exclusive [29, 30]. However, binding of NF-κB is more efficient than NFAT since it is enhanced by cooperative interactions with Sp1 [31]. Although mutation of the NF-κB sites results in only a modest inhibition of virus growth in most transformed cell lines [32], signaling through the viral enhancer is essential in order to re-activate latent proviruses and support virus replication in primary T-cells, regardless of whether it is stimulated by NF-κB or by NFAT [33-37].

## ELONGATION CONTROL OF HIV TRANSCRIPTION BY TAT

The HIV promoter is distinct from cellular promoters because it is highly dependent upon the viral trans-activator protein Tat. The first evidence that HIV transcription is dependent upon a viral factor came from experiments by Sodroski *et al*. [38, 39] who noted that the expression of reporter genes placed under the control of the viral long terminal repeat (LTR) was dependent upon a *trans*-activating factor (Tat) present in HIV-infected cells. Deletion analysis of the viral LTR showed that Tat activity required the transactivation-responsive region (TAR), a regulatory element located downstream of the initiation site for transcription between nucleotides +1 and +59 (Fig. **1**). The interactions between Tat and TAR are direct: Dingwall *et al*. [40-41] demonstrated that Tat is able to specifically recognize TAR RNA and mapped its recognition site to a U-rich bulge near the apex of the TAR RNA stem. Detailed analysis of Tat’s interactions with TAR RNA by NMR subsequently revealed that Tat recognition of TAR requires conformational changes in the RNA structure involving the displacement of the first residue in the bulge (U23) by one of the arginine side chains present in the basic binding domain of the Tat protein to create a binding pocket together with the adjacent G26:C39 base pair [42-45].

## P-TEFB IS AN ESSENTIAL COFACTOR FOR TAT MEDIATED TRANSCRIPTIONAL ELONGATION

The use of a transcribed RNA element to regulate HIV transcription suggested that Tat might be regulating HIV-1 transcriptional elongation, rather than transcriptional initiation. Early RNase protection experiments by Kao *et al*. [46] demonstrated that in the absence of Tat, the majority of RNA polymerases initiating transcription stall near the promoter, whereas in the presence of Tat there is a dramatic increase in the density of RNA polymerases found downstream of the promoter. Promoter proximal pausing has now been recognized as a common feature of many cellular genes, but at the time of the initial discovery in HIV it was unprecedented [47-50].

A 10-year long search for cellular cofactors required for Tat activity culminated with the identification of the elongation factor P-TEFb in 1997 [51-52]. P-TEFb was originally identified as global transcription elongation factor that was selectively inhibited by an ATP analog, DRB (5,6-Dichlorobenzimidazole 1-β-D-ribofuranoside)without directly blocking RNA polymerase II (RNAP II) activity [53,54]. Since DRB also selectively blocks the ability of Tat to stimulate productive elongation, it was proposed that the elongation activity of Tat on the proviral DNA has to be mediated by a DRB-sensitive host elongation factor [55]. Subsequently Herrmann and Rice used HIV Tat as bait to affinity purify a DRB-sensitive Tat-associated kinase (TAK) from HeLa nuclear extracts which could hyperphosphorylate the C-terminal domain of the large subunit RNAP II [56]. TAK was unequivocally identified as P-TEFb when Zhu *et al*. [52] cloned and identified the kinase subunit, CDK9 [53, 57]. Simultaneously, a set of novel CDK9 protein kinase inhibitors were shown be selective inhibitors of HIV-1 transcription [51].

In parallel, the search for cellular factors capable of interacting with TAR RNA also pointed to P-TEFb as a critical co-factor for Tat activation of elongation. Wei *et al*., [58] discovered that P-TEFb contains a cyclin component, CycT1, which can form a stable complex with CDK9, Tat and TAR RNA. Crucially, for a putative Tat co-factor, complex formation between Tat, P-TEFb and TAR requires both the Tat binding site and the essential TAR RNA loop sequence.

After these seminal biochemical observations, persuasive genetic evidence demonstrated that CycT1 is essential for Tat activity. The murine CycT1 sequence differs from the human sequence by a single substitution of cysteine 261 for tyrosine and is unable to interact with HIV-1 Tat. Introduction of Y261 into the human CycT1 blocked HIV-1 transactivation in transfected cells whereas, conversely, introduction of C261 into the murine CycT1 restored Tat-mediated transactivation [59-62].

Human cyclin T1 (hCycT1) has been shown to be the only form of cyclin T that is co-opted by Tat to mediate efficient elongation of HIV [63]. hCycT1 forms the regulatory subunit in a majority of cellular P-TEFb [52, 63], however, as described below, resting T cells can survive when CycT1 levels are absent because they can utilize the homologous cyclin T2a and T2b subunits to sustain transcription.

Finally, in an illuminating paper, culminating over 2 decades of research on P-TEFb by David Price and his colleagues, the crystal structure of a Tat:pTEFb complex was determined in 2010 [64]. The structure shows that Tat forms extensive contacts both with the CycT1 subunit of P-TEFb and also with the T-loop of the Cdk9 subunit (Fig. **1**).

## CONTROL OF TRANSCRIPTION ELONGATION BY TAT

**Fig. (3):**
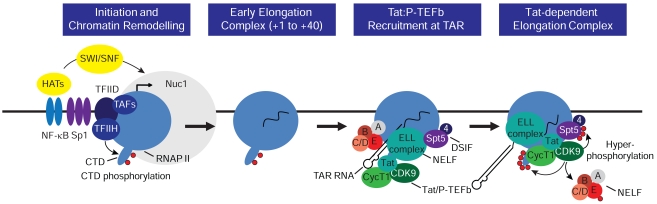
**Activation of HIV transcription elongation by Tat and P-TEFb.** In latent proviruses transcription elongation is very inefficient due to absence of the transcription elongation factor NF-κB as well as chromatin restrictions. Initiation is strongly induced by NF-κB, which acts primarily to remove chromatin restrictions near the promoter through recruitment of histone acetyltransferases and chromatin remodeling factors. After the transcription through the TAR element, both NELF and the Tat/P-TEFb complex (including CDK9 and CycT1 and the accessory elongation ELL2 complex) are recruited to the elongation complex *via* binding interactions with TAR RNA. This activates the CDK9 kinase and leads to hyperphosphorylation of the CTD of RNA polymerase II, Spt5 and NELF-E. The phosphorylation of NELF-E leads to its release. The presence of hyperphosphorylated RNAP II and Spt5 allows enhanced transcription of the full HIV genome.

There is no specific host repressor that directs a provirus to become latent. Instead, the switch between productive transcription and latency is due to the manipulation of the powerful feedback mechanism fueled by Tat (Fig. **1**). In the absence of Tat latently infected cells carry paused transcription complexes near their promoters and accumulate short transcripts that terminate shortly after TAR RNA [65-67]. The promoter-proximal pausing events that need to be overcome by Tat thus represent a critical rate-limiting step in the productive synthesis of HIV mRNA synthesis that is a characteristic of the latent state of the provirus. The detailed transactivation mechanism involves a complex set of phosphorylation events mediated by the Tat-activated P-TEFb that modify both positive and negative cellular elongation factors (Fig. **3**).

Like all cellular genes, HIV transcription initiation is triggered by the phosphorylation of the C-terminal domain (CTD) of the large subunit of RNAP II by the CDK7 subunit of TFIIH at Ser-5 residues of the heptad repeat sequence [68-69]. The nascent transcription complex is able transcribe through the 59-nucleotide TAR RNA hairpin structure before pausing is induced by the negative host elongation factors (NELF) and the DRB sensitivity-inducing factor (DSIF) [70-73]. The Tat/P-TEFb complex cooperatively binds to the nascent TAR RNA bringing the CDK9 kinase of P-TEFb into proximity of the paused RNAP II complex [58, 74].

The binding of Tat to P-TEFb induces significant conformational changes in CDK9 that constitutively activate the enzyme [58, 64, 68] and permit it to extensively phosphorylate multiple proteins in the transcriptional elongation complex. Phosphorylation of the NELF-E subunit by P-TEFb forces dissociation of NELF from TAR and releases paused transcription elongation complexes [73, 75-76]. Cell-free transcription studies have shown that Tat:P-TEFb also hyperphosphorylates the RNAP II CTD during elongation [68, 77]. This reaction creates a novel form of the RNA polymerase that is highly enriched for phosphorylated Ser-2 residues in the CTD and has enhanced processivity [77-78]. Finally, P-TEFb is also able to extensively phosphorylate Spt5, a subunit of DSIF, which carries a CTD homologous to the RNAP II CTD [79-81]. Although the unmodified DSIF inhibits elongation [76], phosphorylation of Spt5 separates it from the rest of the complex and converts it into a positive elongation factor that stabilizes transcription complexes at terminator sequences [79, 82]. Both cell-free and chromatin immunoprecipitation studies have demonstrated that the Tat: P-TEFb complex forms a stable interaction with the RNAP II elongation machinery suggesting that it facilitates transcription elongation at multiple sites along the proviral genome [77, 79, 83-84]. Thus, Tat and P-TEFb are able to stimulate HIV-1 transcription both through the removal of blocks to elongation imposed by NELF and DSIF and by the enhancement of RNAP II processivity through the phosphorylation of Spt5 and the RNAP II CTD.

Recent proteomic studies have shown that in addition to P-TEFb, Tat also helps to direct the transcription factor ELL2 and co-activators belonging to the MLL-fusion protein family (AFF4, ENL, and AF9) to the HIV LTR as part of a so-called super elongation complex (SEC) [85-86]. RNAi knockdown of SEC components dramatically reduces Tat-dependent HIV LTR-driven reporter gene expression, demonstrating a direct role for the SEC in HIV elongation [85-86]. In addition to facilitating the recruitment of the SEC, Tat can also stabilize the SEC complex by preventing the rapid proteasomal degradation of ELL2 [86]. Recently ENL and AF9 were shown to possess a YEATS domain that interacts with the PAF1 subunit of Polymerase-Associated Factor complex (PAFc) [87]. This additional interaction is likely to enhance SEC association with the promoter proximally paused RNAP II complex.

A poorly understood aspect of HIV transcriptional control is the coupling that occurs between transcriptional elongation, the regulation of splicing, polyadenylation and RNA export. Evidence that the splicing-associated c-Ski-interacting protein, SKIP, activates both Tat transactivation and HIV-1 splicing provides an intriguing insight into how these diverse events may be coordinated [88]. Furthermore, P-TEFb kinase activity has been shown to be important in controlling splicing and 3’-end processing of pre-mRNA transcripts [89-92]. In addition the CDK11 kinase, which is related to CDK9, has been recently implicated in control of HIV transcription and processing. Valenti *et al*. [93] found that HIV-1 expression is potently inhibited by set of factors that includes eIF3f, the SR protein 9G8, and CDK11, all of which contribute to HIV mRNA 3’ end processing. Finally the CDK13 kinase has been implicated in the regulation of HIV splicing [94]. Thus, in common with cellular genes, HIV may employ a series of CDK kinases that traverse the genome along with the elongation complex and orchestrate pausing, splicing and termination events by differentially phosphorylating the RNAP II CTD [95].

## POSITIVE FEEDBACK CONTROL OF HIV TRANSCRIPTION BY TAT DETERMINES PROVIRAL LATENCY

The strong amplification of transcription stimulated by Tat, coupled with the disproportionate decline in transcription that ensues when Tat levels become restricted, gives the HIV promoter a “bipolar” character: in the absence of Tat transcription is more restricted than from a typical cellular promoter, whereas in the presence of Tat, the activated complex produces unprecedented levels of viral transcripts. Insightful studies by Weinberger *et al*. [96-98] and Burnett *et al*. [99] have emphasized how stochastic fluctuations in Tat gene expression can act as a molecular switch forcing the virus into a Tat-dependent “on state” or a Tat-independent “off state”.

An important consequence of the bipolar character of the HIV promoter is that small changes in initiation rates are sufficient to restrict Tat production and lead to enhanced rates of viral entry into latency (Fig. **1**). This switching mechanism crucially depends on the auto-regulation and activity of Tat. The introduction of mutations into the NF-κB and Sp1 binding sites, which reduce initiation [99], and mutations that attenuate Tat activity both lead to an increased frequency of viruses entering latency [100]. Similarly, viruses recovered from the latently-infected CD4^+^ T cells of patients are enriched for HIV-1 Tat variants with impaired transactivation activity [101]. The importance of the feedback mechanism is demonstrated by the observation that expression of Tat *in trans* from an ectopic promoter, results in constitutive activation of HIV proviruses that are unable to enter latency [100]. Similarly, changes in the cellular environment that restrict transcription initiation are able to reduce Tat availability and force the virus into latency, but the virus remains poised to resume its replication in response to triggers that stimulate transcription initiation and restore Tat levels. Because of the Tat feedback mechanism, when latently infected cells are partially activated, intermediate viral expression levels are rarely observed. Instead, the subset of cells that is able to produce Tat becomes fully activated, while the subset of cells that fails to achieve threshold levels of Tat reverts to a silenced state.

HIV latency can therefore be thought of as a consequence of changes in the cellular environment that lead to reduced Tat levels. At the chromatin level, epigenetic silencing is used to restrict HIV transcription initiation. In addition, several features of the metabolism of resting CD4^+ ^T cells are critical for the establishment of latency. First, resting cells lack the co-activating factors NF-κB or NFAT. Induction of either transcription factor by drugs, or by T-cell receptor activation, provides a powerful signal leading to the resumption of transcription by latent HIV proviruses. Secondly, reductions in the level the P-TEFb component CycT1 and sequestration of the P-TEFb complex by the HEXIM/7SK RNA complex also appear to restrict HIV transcription in resting lymphocytes. In addition to these mechanisms, it has also been suggested that HIV mRNA export is impaired in resting T cells, posing a further barrier to expression of provirus in resting cells. Below we describe these key mechanisms used to limit transcription initiation and Tat-mediated transcription elongation in latently infected cells.

## EPIGENETIC MECHANISMS THAT LIMIT HIV TRANSCRIPTION INITIATION

HIV integrates into the host genome preferentially within actively transcribed intronic regions [102-104]. Following integration a highly ordered nucleosomal structure is assembled surrounding the promoter [105-107]. These nucleosomal structures, especially nucleosome 1 (Nuc-1), which is positioned around the transcription start site and is therefore able to block RNAP II initiation [105], play a crucial role in regulating HIV transcription and contribute to the transcriptional silencing of the provirus by serving as targets for epigenetic modifications (Fig. **2**). Latent HIV proviruses are subject to a wide range of epigenetic silencing mechanisms including restrictions imposed by deacetylated histones [108], methylated histones [109-111] and DNA methylation [112-113].

The first epigenetic modification of HIV that was identified was the repression of HIV transcription by the recruitment of histone deacetylases (HDACs). In a series of elegant mechanistic studies Margolis and colleagues have detailed how the cooperative binding of LSF and YY-1 to the LTR mediates the recruitment of HDAC1 to the Nuc-1 region of the LTR and identified HDACs as critical repressors of HIV transcription [108, 114-116]. Subsequently several additional cellular proteins have been identified that can enhance HDAC recruitment to the HIV provirus. Williams *et al. *[117] found that p50 subunits of NF-κB are constitutively bound to the NF-κB element at the LTR of the latent proviral DNA. NF-κB p50 homodimer occupancy of the LTR mediated the recruitment of HDAC1 leading to HIV repression. This is reversed by displacement of the homodimer by the p65/p50 heterodimer following TNF-α stimulation. HDACs can also be recruited to the HIV LTR by CBF-1, a key regulator of the Notch signaling pathway [35, 118]. Knockdown of CBF-1 by shRNA stimulates the partial reactivation of viral expression that is associated with disappearance of HDAC1 from the LTR, enhanced acetylation of Nuc-1 at histone H3, and improved RNAP II recruitment [118]. In a primary CD4 T cell model of HIV latency, CBF-1 and its associated co-factors CIR and mSin3A are bound at the latent HIV LTR in conjunction with HDAC1 and other markers of restrictive chromatin that are removed upon reactivating these cells through the T-cell receptor [35].

Latent HIV-1 proviruses also carry methylated histone H3 which has been either trimethylated on lysine 9 (H3K9me3) or lysine 27 (H3K27me3) [100, 110, 111] or dimethylated on lysine 9 (H3K9me2) [119]. Each of these modified histones are considered to be repressive marks for cellular genes [120]. SUV39H1, which is the histone lysine methyltransferase (HKMT) responsible for synthesizing H3K9me3, has been implicated in maintaining HIV-1 latency in microglial cells because of its interactions with CTIP-2 and HP1γ [110-111]. In these systems, knockdown of either CTIP-2 or HP1γ proteins led to activation of HIV-1. Similarly, Imai *et al*. [119] have proposed that the HKMT G9a, which is responsible for creating di-methyl H3K9, can also contribute to the maintenance of HIV-1 latency. Our laboratory has recently demonstrated that EZH2, the enzyme responsible for H3K27me3 formation is found at the promoter of latent HIV-1 proviruses in T-cells together with the corresponding H3K27me3 [109]. Knockdown of EZH2 with shRNA, or inhibition of EZH2 with chemical inhibitors, efficiently reactivates a significant portion of silenced proviruses.

In addition to its enzymatic activity, EZH2 acts as a structural component of the Polycomb Repressive Complex 2 (PRC2). Many intriguing parallels between mechanisms associated with PRC2 silencing of genes during early development and HIV transcriptional control suggest that polycomb can be regarded as a central regulator of HIV epigenetic silencing. PRC2 can serve as a binding platform for multiple histone modifying and DNA modifying enzymes including DNA methyltransferase-1 (DNMT1) [121], the SWI/SNF component bromo-domain containing protein Brd7 [122], and histone deacetylases [123]. It seems likely that these additional components of the silencing machinery can also contribute to the development of HIV latency, and some of these factors have already been associated with HIV latency. For example, DNMT1 is known to mediate methylation of the HIV LTR and further enhance HIV-1 latency [112-113, 121]. PRC2 characteristically targets genes that carry paused RNAP II and generate short RNA transcripts, analogously to the HIV provirus [124-125]. It is tempting to speculate that the short HIV transcripts containing TAR RNA facilitate the recruitment of PRC2 to the LTR. In addition, PRC2 selectively targets genes that contain domains of 'bivalent' chromatin (genes carrying both the histone H3K4me3 and H3K27me37 markers) [126-127]. Similarly, we have recently found H3K4me3 is also associated with the HIV provirus, especially in the downstream Nuc-2 region (unpublished data). Finally, PRC2 is typically found at genes that are enriched in CpG islands [126] and HIV also carries CpG islands in its LTR which are subject to DNA methylation [112] and could contribute to PRC2 recruitment.

In contrast to developmentally-regulated cellular genes, where gene silencing is uniform, epigenetic silencing of HIV-1 results in complex and heterogeneous patterns of histone modifications and DNA methylation [100, 112-113, 128]. Heterogeneity of epigenetic markers exists both between individual clones and, more surprisingly, within clonal populations that carry identical integrated proviruses [109, 112-113]. This epigenetic variation may provide an explanation for why certain subsets of silenced proviruses fail to get reactivated when cells are stimulated with exogenous signals and many of the drug candidates currently under evaluation.

## CONTROL OF LATENCY BY TRANSCRIPTIONAL INTERFERENCE

The observation that HIV integrates within highly active transcription units prompted investigations into whether latency arises because of transcriptional interference between the host promoter and the viral LTR. Lenasi *et al*. [129] showed that in latently infected Jurkat T-cell lines, host-initiated transcripts terminated at the polyadenylation site in the 5’ LTR of the integrated proviruses. Similarly, Han *et al*. [130] showed in cell lines that were engineered to insert HIV proviruses in either orientation downstream of the HPRT gene, readthrough transcription inhibited HIV-1 gene expression for convergently orientated provirus but enhanced HIV-1 gene expression when HIV-1 was in the same orientation as the host gene.

Although transcriptional interference was clearly documented in several proviral clones, it remains unclear whether it is a primary cause of latency or a consequence of the insertion of a repressed provirus into an active gene. Duverger *et al*. [131] have argued that transcriptional interference leads to the “silent integration” of proviruses in the majority of latently infected cells. However, these experiments were designed to select for population of viruses that were subjected to immediate silencing events. By contrast, studies from our laboratory have shown that after the selection of cells that carry highly expressed viruses there is progressive silencing due to the imposition of epigenetic restrictions [35, 100, 109]. If transcriptional interference were the dominant mechanism driving HIV latency, then it might be expected that there is a strong orientation bias seen in latently infected cells. Shan *et al*. [103] observed a modest preference for integration in the same transcriptional orientation as the host gene (63.8% *vs* 36.2%) which was not observed in acutely infected or persistently infected cells, suggesting that transcriptional interference can play an important role, but not an exclusive role, in establishing latency.

Recently Gallastegui *et al*. [132] used detailed RNA-PCR assays to analyze transcriptional interference. They found that HIV integration into an intron of a gene does not abolish expression of its normally spliced transcript and host gene expression was decreased when HIV was reactivated, the opposite of what would be expected from a model of transcriptional interference where host gene transcription prevents HIV transcription. A possible link between the observations that HIV chromatin structures are strongly repressive and transcriptional interference mechanisms comes from the observation that depletion of chromatin remodeling factors which can associate with elongating transcription complexes (e.g. Spt6, Chd1, and FACT, or the histone chaperones ASF1a and HIRA), promoted HIV reactivation concomitantly with chromatin relaxation and a decrease in general RNA polymerase activity [132].

## SEQUESTRATION OF TRANSCRIPTION INITIATION FACTORS

The HIV promoter is exquisitely sensitive to the activation state of the infected cell. Activated CD4 T cells, which can be readily and productively infected with HIV, provide a suitable environment for efficient HIV transcription by expressing high nuclear levels of NF-κB or NFAT and AP-1. By contrast, resting memory T cells are characterized by the cytoplasmic sequestration of NF-κB, NFAT, and AP-1 which severely limits transcription initiation. TCR stimulation of resting T cells induces a complex cascade of pathways leading to the activation of NF-κB through a protein kinase C-mediated pathway and NFAT through the Ca^+2^-calcineurin pathway.

NF-κB binding to the HIV LTR triggers proviral reactivation by directing recruitment of the histone acetyltransferases (HATs) to the HIV LTR [133-136]. The acetylation of histones near the HIV promoter in turn provides a signal for the recruitment of the chromatin remodeling complex BAF which activates transcription by displacing the nucleosome-1 (Nuc-1) which is positioned immediately downstream from the transcriptional start site [105-107, 137-139]. The recruitment of HATs may also help to stabilize NF-κB on the viral promoter, since acetylation [140-141] and methylation [142] of the p65 subunit enhances its DNA binding affinity. NFAT also interacts with the HIV LTR *via *the NF-κB binding sites. It seems likely that members of the NFAT family also serve to mediate HAT recruitment to the HIV-1 LTR since they are known to recruit the coactivators p300 and CBP to cellular genes [143].

Although there has been a long standing controversy about whether NFAT [28, 34, 144] or NF-κB [35-36, 145-146] is the dominant factor mediating proviral reactivation in primary CD4^+^ T cells, it is now evident that multiple cellular pathways are able to independently reactivate latent HIV expression. In latently infected primary T cells derived from thymocytes both the PKC pathway leading to NF-κB activation and the NFAT are able to stimulate virus expression [145]. TLR5 stimulation induces activation of NF-κB and can reactivate latent HIV-1 in quiescent central memory CD4+ T cells [147]. Similarly, inducers of NF-κB such as prostratin and HIV-1-reactivating protein factor (HRF) [146, 148-149] are potent inducers of latent HIV proviruses in resting memory T-cells. By contrast, in polarized T-cells generated *in vitro*, NFAT is clearly the exclusive activator of latent proviruses [34]. Thus both transcription factors can stimulate HIV initiation depending on the signaling pathways that have been activated. However, it is unlikely that both transcription factors can act simultaneously since structural studies have shown that both NF-κBand NFAT assume unique, mutually exclusive, conformations upon binding the HIV LTR [29-30, 150-151].

## REGULATION OF P-TEFB

**Fig. (4):**
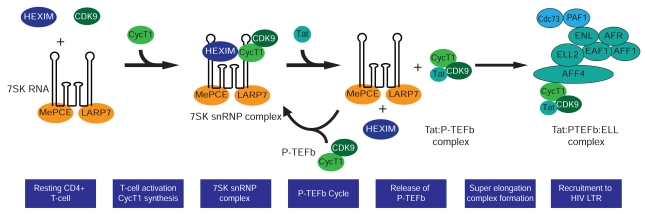
**Model for the activation of P-TEFb in resting CD4^+^ T cells.** In resting CD4^+^ T cells the majority of the P-TEFb in cells there are only low levels of CycT1. Induction of the cells leads to new CycT1 synthesis and the assembly of the transcriptionally inactive 7SK snRNP complex containing 7SK RNA, HEXIM and the RNA binding proteins MePCE and LARP7. Tat disrupts this complex by displacing HEXIM and forming a stable complex with P-TEFb. Prior to recruitment to the transcription complex a larger complex is formed between P-TEFb and transcription elongation factors from the mixed lineage leukemia (MLL) family, including ELL2.

In addition to the transcription initiation factors, P-TEFb is tightly regulated in latently infected T cells (Fig. **4**). First, the expression of hCycT1 in resting CD4^+^ T cells is normally highly restricted. hCycT1 rapidly rise (within 1 h) upon activation of these cells with cytokines, protein kinase C agonists or through the T cell receptor [152-156]. Nuclear factor 90 (NF90), a cellular RNA binding protein, has been recently reported to be an essential factor required for cyclin T1 translation initiation which is upregulated in activated T-cells [157]. Similarly, hCycT1 expression can barely be detected in undifferentiated monocytes due to a microRNA miR-198 that represses hCycT1 protein synthesis [158-159]. Monocyte differentiation into macrophages downregulates miR-198, thereby, permitting hCycT1 expression [158].

There are two isoforms of CDK9 (CDK9_42_ and CDK9_55_) [160]. CDK9_55_ differs structurally from CDK9_42_ by harboring an N-terminal 117-residue extension and is synthesized by a promoter that is upstream of the promoter for CDK9_42 _[160]. Both CDK9_42_ and CDK9_55_ appear to be expressed at similar levels in human peripheral blood lymphocytes [161] and both isoforms possess comparable kinase activity toward the C-terminal domain of the large subunit of RNAP II and can interact with Tat [160-161]. While the basal expression of CDK9_42_ is significantly elevated after ~1-2 days exposure to activation signals there is a reciprocal decrease in the levels of CDK9_55 _[156, 161].

**Fig. (5):**
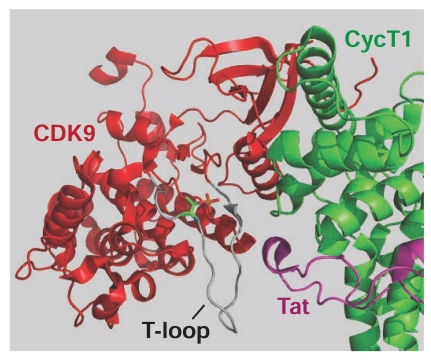
**The CDK9 “T-loop” forms a critical part of the interface between CDK9, CycT1 and Tat.** In this structural model based on Tahirov *et al.* [64] the CDK9 backbone is shown in red, Tat is shown in purple and CycT1 is shown in green. The CDK9 Tloop is highlighted in grey, with the critical phosphorylated T186 residue shown as a side chain. The T-loop is a critical part of the interface between CDK9, CycT1 and Tat. It is believed that alterations in the T-loop geometry induced by post translational modifications and interactions between the three subunits regulates the enzymatic activity of CDK9.

Recent X-ray structures of the P-TEFb heterodimer (CDK9/hCycT1) and the Tat tri-molecular complex (CDK9/hCyT1/Tat) [64] have now defined the interaction interfaces between each of the P-TEFb subunits and Tat (Fig. **5**). The association between CDK9 and hCycT1 triggers the phosphorylation of CDK9 at Thr-186 located within the flexible activation loop (T-loop) of the enzyme, which in turn induces its kinase activity [162]. Tat binds to the phosphorylated P-TEFb and adopts a partially helical secondary structure after forming Zn-coordinated interactions with hCycT1. The Tat/P-TEFb X-ray structure also revealed that the N-terminal region of Tat forms two intermolecular hydrogen bonds with the T-loop region of CDK9 [64].

T-loop phosphorylation of CDK9 is strictly required for P-TEFb molecules to be held within the 7SK snRNP complex; point mutations of Thr-186 that prevent T-loop phosphorylation (both T186A and the phospho-mimetic T186D) inhibit P-TEFb from associating with 7SK snRNA, HEXIM1, and LARP7 [163-164]. It is not entirely clear how CDK9/hCycT1 heterodimerization triggers T-loop phosphorylation of CDK9. Baumli *et al*. [162] have identified Thr-186 to be a potential autophosphorylation site based on mass spectrometry analysis of *in vitro* phosphorylated CDK9. However, a catalytically inactive D167N CDK9 mutant will not only heterodimerize with hCycT1 as efficiently as wild-type, but can also become incorporated into the 7SK snRNP complex [163]. Additionally, *in vitro* kinase and 7SK snRNP reconstitution assays performed by Zhou and colleagues using phosphatase-treated recombinant CDK9 as substrate and HeLa nuclear extracts as the source of kinase activity suggested that there is a novel, still unidentified, nuclear kinase that mediates Thr-186 phosphorylation [165]. It seems likely that both autophosphorylation and external kinases are used to control Thr-186 phosphorylation.

Basal T-loop phosphorylation of CDK9 is extremely low in resting CD4^+^ T-cells [153-154], and this further restricts P-TEFb activity. Concomitant to hCycT1 induction, T-loop phosphorylation is significantly elevated upon brief (within 1 h) stimulation of these cells through the T-cell receptor. By contrast total cellular CDK9 levels are unchanged under these conditions [154].

Transcriptional activity of P-TEFb in peripheral CD4 T cells is further controlled by its sequestration into the 7SK snRNP complex [166-167]. Within this complex 7SK snRNA interacts directly with P-TEFb and its inhibitory protein HEXIM1 and acts as a scaffold to hold the complex together [168-177]. This nuclear regulatory complex also comprises the 7SK snRNA 5’ capping enzyme MEPCE, and LARP7 which binds to the 3’ uridine-rich end of 7SK snRNA and protects it from degradation by nucleases [178-180]. Although P-TEFb molecules that are held within 7SK snRNP are T-loop phosphorylated and therefore catalytically active [163], they are transcriptionally inactive because they are physically unable to be accessed by genes. Nevertheless, P-TEFb-containing 7SK snRNP has been found to be conveniently localized in nuclear speckles in very close proximity to C-terminal hyperphosphorylated RNAP II which would be considered to be a marker of active transcription [152]. A molecular signal or cue that would trigger disassembly of 7SK snRNP and release of P-TEFb is also likely to enable its recruitment to active genes. Therefore, the activation of P-TEFb in resting memory CD4 T cells may require multiple steps involving reversing the restriction on hCycT1 expression, P-TEFb complex formation and T-loop phosphorylation of CDK9, the initial assembly of P-TEFb molecules into 7SK snRNP, relocalization of the inactive complex into nuclear speckles, and the mobilization of active P-TEFb toward transcriptionally active genes (Fig. **4**).

The molecular mechanisms leading to the disassembly of 7SK snRNP and mobilization of P-TEFb toward active genes are not well understood. The bromodomain-containing protein Brd4 is able to remove P-TEFb from the 7SK complex and by virtue of its high affinity interaction with acetylated chromatin, can recruit P-TEFb to active cellular genes [164, 176, 181-182]. Similarly, Tat has also been shown to directly mobilize P-TEFb from 7SK snRNP by outcompeting and physically displacing HEXIM1 from hCycT1 binding [174, 176]. These findings have led to a model that proposes that Tat and Brd4 extract P-TEFb from the inactive 7SK snRNP complex in a mutually exclusive manner.

It seems likely that post-translational modifications of components of the P-TEFb machinery mediate these mobilization events. Recently, we found that T-cell receptor (TCR) signaling in primary CD4 T lymphocytes and Jurkat T cells results in the immediate activation of transcription elongation from latent HIV proviruses, even at times when Tat levels are too low to sustain transcription elongation [35, 84]. This early increase in elongation is due to the activation of P-TEFb by the disruption of 7SK snRNP through the ERK pathway. Natarajan *et al.* also reported that TCR signaling can enhance HIV transcription by stimulating P-TEFb dissociation from the RNP complex [183].

In addition to this physiological pathway a variety of chemical inducers of HIV transcription appear to act by disrupting pTEFb. Zhou and colleagues [165] have reported that treatment of HeLa cells with UV irradiation or the drug HMBA induced 7SK snRNP complex disruption through the activation of two phosphatase enzymes *via *calcium signaling. According to their model, PP2B dephosphorylates HEXIM1 triggering a conformational change within the RNP that exposes the T-loop phosphate of Cdk9 to PP1α. Consequently, this sequential phosphatase activity disassembles the RNP complex. Finally Ott and colleagues [184] have found that the acetylation of hCycT1 by p300 in HeLa cells induces P-TEFb release from 7SK snRNP.

In summary, the data support the model for P-TEFb activation in resting T cells involving both assembly of the 7SK snRNP complex and the subsequent mobilization of P-TEFb by cellular signaling and Tat (Fig. **4**). In the resting T cell there is relatively little P-TEFb assembled into active complexes or the 7SK snRNP complex, primarily because hCycT1 levels are limiting. After activation of the cells, hCycT1 rapidly rise due to new hCycT1 synthesis. The newly produced hCycT1 associates with pre-existing CDK9 and stimulates autophosphorylation of the T-loop and assembly into the 7SK RNP complex. If signaling is sustained, post-translation modifications direct a fraction of the P-TEFb away from the 7SK snRNP complex where it can then bind to Brd4, or if HIV in present, to Tat, and stimulate transcription.

## RESTRICTION OF HIV RNA EXPORT

In addition to the mechanisms affecting transcription described above, post-transcriptional mechanisms may also make a contribution to latency. Using ultra-sensitive methods Lassen *et al*. [185-186] found that purified, primary CD4^+^ T cells derived from patients on HAART have low levels of full-length spliced and unspliced HIV-1 transcripts. Surprisingly both spliced and unspliced HIV-1 RNAs were found in the nucleus suggesting that there may be a block to nuclear mRNA export. This block to HIV-1 RNA export prevents translation of the viral regulatory proteins, thereby further reducing Tat and Rev levels, which contributes to the maintenance of latency.

## PROSPECTS FOR DRUG DISCOVERY

The most practical and effective approach to HIV eradication therapy will be based on small molecule inducers of latent viruses since this type of therapy can be developed using well established drug discovery tools and ultimately therapy can be delivered in normal clinical settings [20, 187-190]. The goal of this therapy is to induce the transcriptional activity of the latent HIV-1 without inducing the polyclonal activation of non-infected cells (a “shock” phase). Once the virus is reactivated a “kill” phase will be used to eliminate the induced cells through existing immune responses, viral cytopathogenicity or cytotoxic drugs. The molecular studies reviewed here also emphasize that HIV has a plethora of potential drug vulnerabilities that could be exploited to prevent its re-emergence from latency. Extensive screens have shown that protein kinase C agonists lacking tumor-promoter activities are a major class of drugs that are able to induce transcriptional activity of the latent HIV-1 without inducing the polyclonal activation of non-infected cells [191]. The range of compounds that are able to activate HIV *via *this mechanism of action is extremely diverse and includes phorboids such as prostratin [146, 149, 156, 192], the clinically-available macrolide bryostatin 1[193-195], and macrocyclic polyesters such as jatrophanes [196]. However, all of these drugs are expected to activate signaling pathways leading to NF-κB mobilization. The second major class of activators are drugs that induce epigenetic modifications such as histone deacetylase (HDAC) inhibitors (SAHA, valproic acid) [197-201] and histone methyltransferase inhibitors (DZNep, BIX01294) [109, 119]. Of all of these compounds SAHA has proven to be the most potent in a variety of cell systems, perhaps because it may also have an impact on P-TEFb levels in resting T cells [202]. Finally new screens are identifying additional drugs with unknown mechanisms, such as disulfiram, that can reactivate HIV in latently infected primary CD4 T cells [37, 188, 203-206].

The detailed molecular studies described above strongly imply that effective activation of the entire latent viral pool may ultimately require a cocktail of drugs that stimulate both transcription initiation and P-TEFb mobilization. It is therefore not too surprising that most of the agents so far identified can therefore only reactivate a subset of latently-infected cells. As insights into HIV latency continue to emerge, it will be important to engage the best researchers from academia and industry in a concerted effort to develop safe and effective HIV eradication strategies.
